# The Chinese Inventory of Psychosocial Balance Short-Form Questionnaire for the Older Adults: Validity and Reliability Study

**DOI:** 10.3389/fpsyg.2022.799967

**Published:** 2022-02-01

**Authors:** Pei-Yun Chen, Wen-Chao Ho, Chyi Lo, Tzu-Pei Yeh

**Affiliations:** ^1^Department of Public Health, China Medical University (Taiwan), Taichung, Taiwan; ^2^School of Nursing, China Medical University (Taiwan), Taichung, Taiwan; ^3^China Medical University Hospital, Taichung, Taiwan

**Keywords:** ego, life span, validity, reliability, elderly

## Abstract

**Background:**

Drawing from Erikson’s theory, Domino and Affonso constructed the Inventory of Psychosocial Balance (IPB), a scale with satisfactory reliability and validity. However, the lack of a credible Chinese version of the scale may hinder research on ego development in Taiwan. The aim of the present study was to construct a short form Chinese IPB. In addition, factor analysis was employed to shorten the original 120-item scale to make it suitable for application in the older adults in the future.

**Methods:**

The study involved three steps: The first step was to establish the 120-items of the Chinese Inventory of Psychosocial Balance (C-IPB), and we conducted translation, back-translation, expert validity, and reliability of pilot study for this step. Following the first step was to construct the short-form C-IPB (CIPB-SF) in the second step, and the CIPB-SF was developed *via* item analysis and factor analysis. Finally, we assessed the reliability and validity of the CIPB-SF *via* structural equation model in the third step.

**Results:**

Three hundred eight older adults without cognitive disorder completed the IPB. The 40-item CIPB-SF was completed through item analysis and factor analysis. The internal consistency test of CIPB-SF and the eight stages were good (Cronbach’s α = 0.81–0.89). The CIPB-SF had acceptable validity, except in the intimacy and identity stages, in which validity was only fair. Compared with the IPB, the CIPB-SF had good reliability and acceptable validity. However, because of its conciseness, the 40-item CIPB-SF was more suited for application among the Chinese elderly population because its application avoids physical overload.

**Conclusion:**

The CIPB-SF served as a concise scale for assessing ego development in our study. This scale can also serve as a useful tool for convenient screening in the future.

## Introduction

The advancement of medical progression increases human longevity, but this does not make death avoidable. Adults’ awareness of death may be the source of anxiousness and stress, especially in the last stages of life in older adults. Accompanying degradation of physiology, the older adults could be aware that their death is approaching, and so appear some negative emotions such as depression (Busch et al., 2018). According to the Erikson theory, while older people in the last stage of life may accept their past and find meanings from their lives, they may reach ego integrity; which means they may face death peacefully (Erikson, 1963).

The psychosocial development in older adults is influenced by their past life experiences (Ardelt et al., 2018). Erikson’s life stage theory of psychosocial development is a rare psychosocial theory that encompasses the entire life span and encompasses eight age-graded stages of ego development, ranging from infancy to late adulthood. According to Erikson’s theory, failure to successfully complete a stage can result in reduce ability to complete subsequent stages, in turn resulting in an unhealthy personality and a sense of self (Erikson, 1963, 1980, 1982). A specific type of crisis occurs in each stage, during which an individual cannot achieve the goal set for each stage (Erikson, 1963, 1982). The task–crisis pairs are as follows: trust vs. mistrust, autonomy vs. shame and doubt, initiative vs. guilt, industry vs. inferiority, identity vs. role confusion, intimacy vs. isolation, generativity vs. stagnation, and integrity vs. despair. The final stage represents the crisis of “integrity vs. despair,” which can be described as the process through which older adults try to find meanings in their lives. Erikson proposed that individuals who had been unsuccessful in resolving earlier crises would face difficulties in resolving later psychosocial crises as well (Erikson, 1963). An individual’s personality subconsciously affects their psychosocial functioning and mental health (Dezutter et al., 2016). Early psychosocial crises may worsen an individual’s depression, self-neglect tendencies, and morbidity. Furthermore, early psychosocial crises may negatively influence the well-being of the older adults (Cuijpers et al., 2013; Saint Onge et al., 2014). On the contrary, by improving this, ego-integrity can be achieved successfully aging and a better quality of life (QOL; Min, 2020). The ego integrity in older people is important; however, a valid measure tool is deficient in Taiwan; the evaluation of ego integrity and related research in older adults is limited.

The measurement tools which include “The Northwestern ego-integrity scale (NEIS)” (Westerhof et al., 2017), “ego-integrity/despair scale” “(Van Hiel and Vansteenkiste, 2009),” and the “inventory of psychosocial balance (IPB)” “(Domino and Affonso, 1990)”were developed according to Erikson theory and comprehensively used in ego integrity in older adults. NEIS includes two subscales (ego-despair and ego-integrity) with 15 items. The higher item mean scores indicate more despair and ego-integrity, respectively. The Cronbach α of NEIS is 0.74 in ego integrity and 0.75 in despair (Westerhof et al., 2017), and it has been applied in many studies (Kleijn et al., 2016; Westerhof et al., 2017).

Ego-integrity/despair scale, developed by Van Hiel and Vansteenkiste (2009), is a 5-point Likert scale, the scores range from 1 (Completely not true) to 5 (Completely true). This scale included ego-despair and ego-integrity sub-scale with 3 items, respectively. The higher mean scores indicated more despair and ego-integrity, and the Cronbach’s alphas were 0.80 in ego integrity and 0.85 in despair (Van Hiel and Vansteenkiste, 2009); it has been used in several studies (Dezutter et al., 2016; Derdaele et al., 2019).

Drawing from Erikson’s theory, Domino and Affonso (1990) constructed the IPB, which has good reliability (Cronbach’s α ranging from 0.64 to 0.79.) and validity and has been applied in many studies (Hannah et al., 1996; Brennan and MacMillan, 2006; Beaumont and Pratt, 2011). IPB covers eight stages of Ericson theory, and each stage contains 15 items (120 items in total); the items were responded in 5-point Likert scale. The higher scores represent better self-development in certain stage (Domino and Affonso, 1990).

Although these three measurement tools all possess good validity and reliability in many studies, a credible Chinese version of these tools in ego development is lacking in Taiwan. Therefore, developing a Chinese version of the scale for ego development measurement is necessary.

The NEIS and ego-integrity/despair scale only include the older adult stage, whereas the IPB covers all eight stages of self-development. The Erikson theory is an important and rare psychological theory because it involves all stages in life, which is valid in older adult mental development. The IPB could be applied in various research more comprehensively; therefore, this research selected IPB to develop a Chinese tool. However, the original version contains 120 items which may be difficult for older adults to complete the questionnaire. Therefore, developing a short version IPB in Chinese is considered cautious and necessary. The aim of this study was to develop a short version Chinese IPB.

## Study Measures

Survey tools in this study included sociodemographic characteristics (age, gender, marital status, religious, self-perceived economic situation, and educational level), IPB, and MOS 36-item short-form health survey (SF-36).

### Inventory of Psychosocial Balance

The IPB is a scale that draws from Erikson’s ego development theory (Domino and Affonso, 1990). This scale initially had 346 items determined with reference to relevant studies and was developed to reflect both positive and negative aspects of the eight stages. The responses are recorded on a 5-point Likert scale, ranging from strongly agree to strongly disagree, and are scored between 1 and 5 points. Five psychologists familiar with Erikson’s theory reviewed each item for clarity of meaning and identified each item’s relevance to a specific stage, after which they wrote written descriptions of each life stage, summarized on the basis of Erikson’s writings. A total of 208 of the original 346 items survived this first step (Domino and Affonso, 1990).

A total of 528 subjects, including students, adults, older adults, and retired adults, were included, and their age ranged from 21 to 88 years. The second process was factor analysis. The factor loading had to be at least 0.3, and the items had to significantly (*p* < 0.01) correlate with the appropriate self-rating. Finally, the 15 best items were selected (Domino and Affonso, 1990). The IPB thus obtained had good reliability demonstrated through internal consistency, evident in a Cronbach’s α ranging from 0.64 to 0.79. The 4–5 week test–retest reliability ranged from 0.79 to 0.90 (Hannah et al., 1996). The validation of the IPB was compared with that of the California Psychological Inventory (CPI)–social maturity index. The results highlighted that six of the eight IPB scales demonstrated positive significant correlations with the CPI–social maturity index, with only the autonomy and intimacy scales exhibiting non-significant coefficients (Hannah et al., 1996).

### MOS 36-Item Short-Form Health Survey (SF-36)

The SF-36 is a 36-item scale to assess the health-related QOL, with higher scores indicating better QOL. The SF-36 involved eight subscales: physical functioning (PF), role physical (RP), bodily pain (BP), general health (GH), vitality (VT), social functioning (SF), role emotional (RE), and mental health (MH). Through applying special algorithms, these eight subscales could contribute to a physical dimension called the physical component scale (PCS), and a mental dimension called the mental component scale (MCS) (Sherbourne and Ware, 1992).

The SF-36 Taiwan version was a popular scale in which internal reliability has reached acceptable level for all scales (α> 0.70). The Cronbach’s α of eight subscales ranged from 0.65–0.92 (PF = 0.91, RP = 0.92, BP = 0.76, GH = 0.82, VT = 0.79, SF = 0.65, RE = 0.87, and MH = 0.78). In terms of validation, each item showed item-scale correlation higher than 0.4. Except item 5 in MH, every item had higher correlation coefficient with its own subscale than other subscales. This indicated good discriminant validity. SF-36 has Taiwanese version and is used commonly in Taiwan for years. The item-scale correlation coefficients of SF-36 range from 0.40 to 0.83 and the rest of the scales have passed the tests of item discriminant validity, except for MH5 (Lu et al., 2003; Tseng et al., 2003).

## Methodology

**FIGURE 1:**
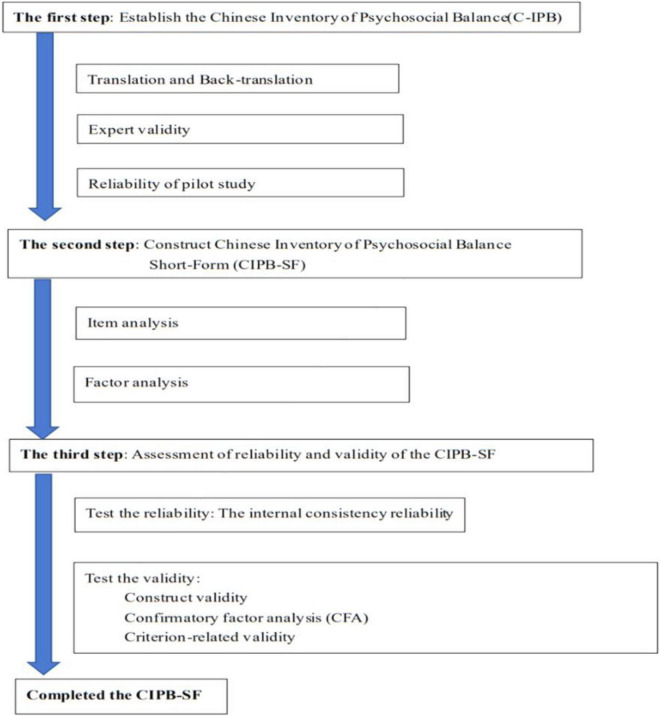
Development and establishing the CIPB-SF.

This study was approved by an institutional review board of China Medical University in Taiwan (Approval No. DMR95-IRB-144). There are various methods for crosscultural adaptation of a questionnaire, but none is considered as the gold standard (Epstein et al., 2015). This study integrated the research methods of Beaton et al. (2000) and Rived-Ocaña et al. (2020) and the steps are shown in Figure 1.

The first step was to develop the 120 items of the Chinese IPB (C-IPB). The process of translation and back-translation was used, and expert validity and reliability test in a pilot study were done.

The second step was to construct C-IPB short-form (CIPB-SF). This step involved item analysis and factor analysis. The item analysis was used to delete those items without discrimination and with poor homogeneity. The left items were tested in factor analysis, and items which showed factor loading more than 0.55 remained, and the initial CIPB-SF was formed.

Finally, the reliability and validity of the CIPB-SF were examined. The reliability was tested in internal consistency, and the convergent validity, confirmatory factor analysis (CFA), and criterion-related validity were done.

### The First Step: Establish the Chinese Inventory of Psychosocial Balance

After obtaining permission from the author of the original IPB questionnaire, the questionnaire was translated following the process recommended by Beaton et al. (2000) and Rived-Ocaña et al. (2020). The first step included “translation, back-translation,” “expert validity,” and “reliability of pilot study.”

#### Translation, Back-Translation

The first translator was a professional translator with a master’s degree and experience in English-to-Chinese translation. After the English-to-Chinese translation, another professional translator back-translated the first Chinese version of the IPB into English. Both translators were Chinese, and they did not engage in any discussions with each other. Thus, both professional translators produced translations of the IPB independently.

#### Expert Validity

Subsequently, expert validity was done. The expert was an academic scholars or a clinical staff with a master or doctoral degree and specialized in psychological or clinical care. We invited five experts (a doctoral degree of nursing, a master degree of psychology, a doctoral degree of social work, a master degree of nursing, and master degree of nursing supervisor) who reviewed both the versions and offered suggestions regarding the questionnaire content, semantics, structure, syntax, orthographic revision, and appropriateness of the translation. They were anonymized to each other during the process. The content validity index (CVI) was used in assessing the content validity. The CVI was a widely useful approach for content validity in instrument development and it could be computed *via* the item-CVI (I-CVI) and the scale-level-CVI (S-CVI). The experts were asked to rate instrument items on a 4-point ordinal scale [4(highly relevant), 3(quite relevant), 2(not relevant), 1(extreme not relevant)]. Item-CVI (I-CVI) is computed as the number of experts who score a rating of 3 or 4 point of each item, divided by the total number of experts. The scale-level CVI (S-CVI) is defined as “the proportion of items on an instrument that achieved a rating of 3 or 4 by the content experts” (Beck and Gable, 2001). After integrating the opinions from five experts, the corrections were done and the revised IPB was returned to the experts for second review. Until every expert agreed the contents of translated IPB, the 120-item C-IPB was completed.

#### Reliability of Pilot Study

Following the expert validity, a pretest was conducted to identify the problems that could be encountered while using the scale in the study and to use the responses obtained during the pretest for consultation purposes in the future. During the pretest, a majority of the participants expressed that filling out the 120-item questionnaire took them 30–45 min and that the long questionnaire tired them. To facilitate economic efficiency and widespread usage, we developed a short form of the C-IPB, called the CIPB-SF.

### The Second Step: Constructing Chinese Inventory of Psychosocial Balance Short-Form

In the second step, the CIPB-SF was developed *via* item analysis and factor analysis.

#### Item Analysis

Item discrimination and a test of homogeneity were used to perform the item analysis. Theoretically, items in a scale should have the capability to distinguish good from bad condition of subjects and those items in the same stage had the same characteristics. Therefore, according to the score on C-IPB, we classified the top 27% of the sample as the high group and the bottom 27% as the low group, and *t*-test was conducted to test if there were differences between the high group and the low group. The items which were not statistically significant implied that the items lacked discrimination and had to be deleted. The homogeneity test was analyzed using correlation coefficients. Correlation coefficients with magnitudes less than 0.3 had little correlation with other items in the same stage. Items were deleted when the correlation coefficients were less than 0.3 (Williams et al., 2010). Based on the above item analysis, the items with discriminative and homogeneous of the scale were entitled in the factor analysis.

#### Factor Analysis

Before factor analysis, we performed Bartlett’s test of sphericity and the Kaiser–Meyer–Olkin measure of sampling adequacy test (KMO test). The method of principal component analysis and varimax rotation were used. The criterion of eigenvalues > 1 was employed to select components (Munro, 2005). Items with factor loadings that exceeded 0.55 were included (Williams et al., 2010; Demoulin, 2016). Finally, the CIPB-SF was established.

### The Third Step: Assessment of Reliability and Validity of the Chinese Inventory of Psychosocial Balance Short-Form

In the third step, we assessed the reliability and validity of the CIPB-SF. We calculated the Cronbach’s α to measure the internal consistency reliability and compare the Cronbach’s α between the CIPB-SF and the C-IPB. When the Cronbach’s α scores were > 0.70, the reliability was considered to be high. The Cronbach’s α scores between 0.35 and 0.7 indicated acceptable reliability (Taber, 2018).

Furthermore, we tested the construct validity, CFA, and criterion-related validity of CIPB-SF.

“Convergent validity” is one of the construct validity and which states that the items having the similar constructs should be highly correlated. One of the method is calculating the correlation coefficients between tools’ subdomains that are considered to measure the same construct (Gregory, 2004).

We tested the “CFA” of CIPB-SF *via* structural equation modeling (SEM). Based on the literature, it is suggested that if the sample size over 500 would be more adequate to use the maximum likelihood (ML) method for estimation (Long, 1997). Due to the sample size in the research was only 308 and the questionnaires were scored as continuous variables, the generalized least square (GLS) method was conducted in the study (Carroll and Ruppert, 1982).

In order to evaluate the “criterion-related validity” of CIPB-SF, we planned to compare the CIPB-SF and SF-36 *via* Pearson’s correlation. The original scale, IPB, was compared with the social maturity index for testing the criterion-related validity of IPB (Domino and Affonso, 1990). Though, the Chinese-form social maturity index does not exist, we still have to evaluate the CIPB-SF with the similar concept and Chinese-form scale. Theoretically, participants with better ego development will have higher scores in QOL (Kleijn et al., 2016). Therefore, we compared CIPB-SF and QOL through Pearson correlation to analyze the criterion-related validity of CIPB-SF.

## Results

### General Information of Participants

Since the Erikson’s theory was applied for the research, the elder adults were qualified for all the eight stages of ego development only. Residential older adults older than 65 years old were contacted by a student pursuing a master’s degree in nursing. Since our study concerned general older adults, we wished to recruit healthy Taiwanese older adults who were also independent. Therefore, the data collection sites were settled to public places. Individuals with dementia or other geriatric cognitive disorders and those who lived in long-term care facilities were not eligible.

Participants were approached by a researcher who explained the study purpose and content, and they signed informed consents before data collection. The mini-mental state examination (MMSE) was developed in 1975 by Folstein and Mc Huge, and the evaluating dimensions included orientation to time and place, registration, attention and calculation, recall, language, repetition, and complex commands. The total score is 30, and a higher value of MMSE indicates a better recognition. MMSE score less than 23 means mild cognition impairment. MMSE is the most used measurement tool in cognitive functions in clinical settings (Galea and Woodward, 2005; Finney et al., 2016). This study applied the MMES as the screening tool, and MMSE scores under 23 are represented as the cut-off score for cognitive impairment. Those participants who had MMSE scores below 23 were excluded in the data analysis (Galea and Woodward, 2005).

A total of 308 questionnaires were retrieved. Participants’ mean age was 71 years (standard deviation = 6.4). In addition, 52.3% of them were women and 96.4% were married. Regarding education levels, 18.5% of participants were uneducated, 37.3% have received high school education or above. In terms of marriage, only 11 participants were never married. Most participants were married or divorced or widowed. There were 52.9% participants who were religious, and 63% participants felt their economic statuses were ordinary. In Table 1, the participants’ socio-demographic characteristics are presented.

**TABLE 1:** Participant socio-demographic characteristics (*N* = 308).

		Mean	*SD*	n	Percentage
Age		71	6.4		
Gender					
	Male			147	47.7
	Female			161	52.3
Education					
	Uneducated			57	18.5
	Elementary and junior			136	44.2
	High school graduate or above			115	37.3
Marriage					
	Yes			297	96.4
	Never			11	3.6
Religious					
	Yes			163	52.9
	No			145	47.1
Self-perceived economic situation					
	Poor			37	12.0
	General			194	63.0
	Rich			25	8.1
	Rejection			52	16.9

### Establish the Chinese Inventory of Psychosocial Balance

After translation and back-translation, the translated Chinese version IPB and English version IPB were dispatched to five experts for check and reviewing. The content, semantics, structure, syntax, orthographic revision, and appropriateness of the translated IPB were carefully examined and considered with revision suggestions. After the first-time revision, the revised Chinese IPB was returned to the experts for further review. When consensus was reached, the CVI was calculated.

The CVI for the C-IPB was 0.7, indicating the scale’s acceptable expert validity (Polit and Beck, 2004). After conducting a final review for content validity, all experts agreed with the scale’s items. Thus, the 120-item scale, C-IPB, was completed.

A pretest was conducted to identify the problems that could be encountered while using the scale in the study and to use the responses obtained during the pretest for consultation purposes in the future. We included 32 subjects who were ≥ 65 years and without any cognitive impairment in the pretest. The pretest results revealed the following: (1) The reliability of the C-IPB was good (Cronbach’s α = 0.82). (2) The length of the 120-item C-IPB was too long for the elderly participants to complete. Many participants mentioned that completing the 120-item C-IPB tired them. Therefore, we decided to shorten the C-IPB on the basis of the pretest results to increase its usefulness.

The C-IPB possessed good reliability with Cronbach’s α = 0.82 and face validity CVI = 0.7; in the meanwhile, we found that completing 120 items is a burden to participants; thus simplifying the C-IPB was necessary.

### Construct Chinese Inventory of Psychosocial Balance Short-Form

#### Item Analysis of the Chinese Inventory of Psychosocial Balance

A method for the comparison of extreme groups was tested. Items 10, 12, 17, 49, 74, 76, 83, 87, and 117 were not statistically significant, implying that those items lacked discrimination and had to be deleted. The remaining 109 items possessed discrimination.

The homogeneity test was analyzed using correlation coefficients. On the basis of the homogeneity test results, 74 items were deleted from the following stages because of having correlation coefficients below 0.3: trust stage (eight items deleted), autonomy stage (10 items deleted), initiative stage (nine items deleted), industry stage (nine items deleted), identity stage (10 items deleted), intimacy stage (nine items deleted), generativity stage (10 items deleted), and integrity stage (nine items deleted).

The result indicated that items 10, 12, 17, 49, 74, 76, 83, 87, and 117 lacked both discrimination and homogeneity and had to be deleted. A total of 46 items were reserved through item analysis and were included in factor analysis.

#### Factor Analysis of the Chinese Inventory of Psychosocial Balance

Before factor analysis, we performed the KMO test. The KMO test results for the C-IPB was 0.86, which indicated that sampling is adequate (Kaiser and Rice, 1974). Through principal component analysis and varimax rotation, eight factors could be extracted from the ego development. Items with factor loadings that exceeded 0.55 were included (Williams et al., 2010; Demoulin, 2016). Table 2 presents the factor analysis results. Finally, we retained five items of each stage in order to avoid confusion for the user in the future.

**TABLE 2:** Factor analysis of C-IPB (*N* = 308).

Component	Factor loading							
	Factor1	Factor2	Factor3	Factor4	Factor5	Factor6	Factor7	Factor8
***“Trust vs. Mistrust”***								
9. I have confidence in my own abilities	**0.73**	0.63	0.00	0.12	0.23	0.32	0.05	0.00
41. Most conflicts between people can be resolved by discussion	**0.72**	0.67	0.05	0.04	0.01	0.09	0.03	0.17
113. Basically, I think I am an all right person	**0.64**	0.50	0.02	0.23	0.27	0.27	0.04	0.11
97. In general I am optimistic person	**0.58**	0.21	0.27	0.13	0.21	0.21	0.18	0.05
33. Suffering can be meaningful for the growth of person	**0.57**	0.10	0.32	0.27	0.02	0.20	0.04	0.01
105. People have the capacity to solve their problems	**0.57 (delete)**	0.48	0.04	0.14	0.08	0.10	0.17	0.33
*89. I find I am open to new ideas	**0.54 (delete)**	0.48	0.21	0.01	0.22	0.02	0.11	0.03
***“Autonomy vs. Shame and doubt”***								
114.“A place for everything and everything in its place” is my motto	0.15	**0.78**	0.54	0.03	0.05	0.10	0.10	0.15
34. I am a very organized person	0.15	**0.71**	0.02	0.08	0.20	0.56	0.26	0.12
82. We would all be better off if people obeyed the laws we have	0.16	**0.70**	0.30	0.14	0.00	0.58	0.00	0.03
66. It is important for young people to be independent	0.02	**0.59**	0.09	0.47	0.23	0.38	0.13	0.00
58. I am quite self-sufficient	0.51	**0.58**	0.26	0.25	0.10	0.20	0.09	0.14
***“Initiative vs. Guilt”***								
107. It’s easy for me to begin new projects	0.18	0.04	**0.71**	0.54	0.14	0.26	0.18	0.01
115. I am a highly curious individual	0.05	0.07	**0.65**	0.05	0.46	0.33	0.13	0.12
51. As a child I often took things apart to see how they worked	0.19	0.02	**0.65**	0.34	0.23	0.30	0.27	0.10
67. I would love to invent a new way of doing something	0.02	0.09	**0.61**	0.23	0.41	0.23	0.05	0.10
99. Friends and acquaintances often tell me that my ideas are original	0.29	0.11	**0.60**	0.25	0.24	0.24	0.22	0.10
3. I am easily embarrassed	0.10	0.11	**0.59 (delete)**	0.16	0.48	0.08	0.20	0.24
***“Industry vs. Inferiority”***								
116. I genuinely enjoy work	0.49	0.13	0.07	**0.72**	0.02	0.16	0.01	0.09
108. I like to be busy	0.01	0.34	0.20	**0.71**	0.09	0.46	0.01	0.10
92. When necessary, I can devote a lot of energy to a task	0.21	0.11	0.10	**0.70**	0.45	0.07	0.15	0.14
28. Others would describe me as a productive person	0.49	0.01	0.09	**0.67**	0.02	0.16	0.13	0.07
52. Work brings me great satisfaction	0.05	0.19	0.04	**0.67**	0.45	0.01	0.04	0.04
4. Most people around me seem to be more talented than I am	0.35	0.23	0.01	**0.62 (delete)**	0.14	0.27	0.52	0.03
***“Identity vs. Role confusion”***								
69. I feel very comfortable with the value I have	0.21	0.33	0.14	0.02	**0.75**	0.14	0.04	0.02
5. Sometimes I wonder who I really am	0.05	0.39	0.07	0.16	**0.65**	0.07	0.07	0.09
109. Friends would describe me as a very changeable person	0.52	0.27	0.02	0.15	**0.62**	0.20	0.12	0.10
93. My adolescence was fairly stormy	0.20	0.14	0.14	0.09	**0.58**	0.13	0.37	0.35
101. There are times when I wish I had been born of the opposite sex	0.12	0.26	0.21	0.11	**0.55**	0.15	0.27	0.02
***“Intimacy vs. Isolation”***								
46. There have been people in my life with whom I have been willing to share my innermost thoughts	0.04	0.12	0.23	0.12	0.09	**0.66**	0.49	0.28
86. Overall, my sexual life has been satisfactory	0.41	0.32	0.08	0.05	0.12	**0.66**	0.47	0.14
102. When I was a teenager I had a very close friend with whom I shared many experiences	0.13	0.09	0.41	0.36	0.14	**0.66**	0.30	0.27
38. There have been times when I felt extremely close to someone I loved	0.26	0.00	0.39	0.15	0.06	**0.60**	0.20	0.28
6. I have experienced some very close friendships	0.16	0.29	0.11	0.33	0.20	**0.58**	0.18	0.41
110. I enjoy being with people	0.05	0.26	0.17	0.17	0.02	**0.55 (delete)**	0.54	0.03
***“Generativity vs. Stagnation”***								
119. I enjoy learning new skills	0.06	0.06	0.07	0.27	0.11	0.09	**0.79**	0.42
47. To be a good parent is one of the most challenging tasks people face	0.13	0.07	0.24	0.16	0.17	0.18	**0.68**	0.52
39. If it were possible, I would greatly enjoy teaching adolescents	0.15	0.08	0.27	0.17	0.25	0.09	**0.62**	0.53
63. I am very concerned that our children will grow in a polluted world	0.15	0.07	0.17	0.16	0.06	0.09	**0.60**	0.42
7. I derive great pleasure in watching a child master a new skill	0.13	0.10	0.29	0.15	0.18	0.13	**0.57**	0.53
***“Integrity vs. Despair”***								
56. There are many things I enjoy in life	0.53	0.08	0.21	0.08	0.10	0.29	0.10	**0.75**
40. Life has been good to me	0.01	0.49	0.11	0.21	0.35	0.08	0.02	**0.74**
48. I have left my mark on the world	0.47	0.15	0.36	0.20	0.07	0.09	0.05	**0.71**
64. I find little sense in living	0.40	0.48	0.05	0.07	0.16	0.05	0.17	**0.59**
96. When I die I will be missed	0.47	0.12	0.06	0.09	0.06	0.23	0.22	**0.57**
104. I have given serious thought to the meaning of life	0.16	0.20	0.10	0.10	0.18	0.53	0.06	**0.56 (delete)**

*The bold values represented the factor loading values in the same group for better readibility.*

### Assessment of Reliability and Validity of the Chinese Inventory of Psychosocial Balance Short-Form

#### Reliability Coefficients for the Chinese Inventory of Psychosocial Balance Short-Form

Table 3 presents the results. The reliability of the CIPB-SF was found to be adequate, with internal consistency and a Cronbach’s α = 0.85. Compared to the CIPB-SF and the C-IPB, the Cronbach’s α of each stage was similar. The Cronbach’s α of each stage ranged from 0.81 to 0.89 of the CIPB-SF, and from 0.70 to 0.77 of the C-IPB. Table 3 showed the total score and subscores of the eight stages. All stages exhibited sufficient internal consistency, as indicated by good Cronbach’s α coefficients. The highest Cronbach’s α coefficient was 0.89 in the identity stage, and the lowest was 0.81 in the trust and integrity stages.

**TABLE 3:** Reliability coefficients (Cronbach’s α) for the CIPB-SF and C-IPB (*N* = 308).

	CIPB-SF	C-IPB
Stage_1	0.81	0.70
Stage_2	0.82	0.71
Stage_3	0.84	0.77
Stage_4	0.82	0.76
Stage_5	0.89	0.77
Stage_6	0.85	0.76
Stage_7	0.82	0.70
Stage_8	0.81	0.72
Eight stages	0.85	0.74

*Stage_1: The first stage (trust vs. mistrust); Stage_2: The second stage (autonomy vs. shame and doubt).*

*Stage_3: The third stage (initiative vs. guilt); Stage_4: The fourth stage (industry vs. inferiority).*

*Stage_5: The fifth stage (identity vs. role confusion); Stage_6: The sixth stage (intimacy vs. isolation).*

*Stage_7: The seventh stage (generativity vs. stagnation); Stage_8: The eighth stage (integrity vs. despair).*

*Eight stage: A composite score of the eight subscales.*

#### Criterion-Related Validity of Chinese Inventory of Psychosocial Balance Short-Form

We evaluated the Pearson’s correlation coefficient between the CIPB-SF and SF-36 (Table 4). The CIPB-SF subscales all had a significant positive relation (*p* < 0.001) to the MCS of SF-36. The results indicated that the better ego development status the individuals have, the better mental dimension of QOL they will have. The result was in conformity with the hypothesis (Kleijn et al., 2016), and it means the CIPB-SF had a good criterion-related validity.

**TABLE 4:** Correlation among each item of subscale, all stages in CIPB-SF, and MCS (*N* = 308).

Stage	MCS	Eight stage	Stage_1	Stage_2	Stage_3	Stage_4	Stage_5	Stage_6	Stage_7	Stage_8
Item	R	r	r	r	r	r	r	r	r	r
**Stage_1**	0.47**									
9		**0.62**	0.72	0.51	0.50	0.62	0.04	0.29	0.43	0.49
41		**0.50**	0.56	0.54	0.24	0.46	0.06	0.26	0.51	0.33
113		**0.64**	0.69	0.57	0.36	0.50	0.15	0.40	0.50	0.54
97		**0.46**	0.57	0.31	0.43	0.34	−0.06	0.39	0.43	0.28
33		**0.53**	0.59	0.36	0.36	0.49	0.19	0.27	0.26	0.52
**Stage_2**	0.43**									
114		**0.51**	0.50	0.69	0.33	0.51	0.02	0.21	0.42	0.39
34		**0.52**	0.47	0.63	0.26	0.45	0.29	0.16	0.29	0.51
82		**0.43**	0.41	0.61	0.15	0.39	0.24	0.11	0.31	0.36
66		**0.53**	0.46	0.69	0.29	0.40	0.11	0.33	0.50	0.36
58		**0.60**	0.54	0.75	0.43	0.54	0.09	0.28	0.51	0.44
**Stage_3**	0.24**									
107		**0.46**	0.36	0.36	0.61	0.42	−0.07	0.33	0.39	0.32
115		**0.41**	0.29	0.27	0.67	0.20	−0.01	0.42	0.35	0.25
51		**0.41**	0.35	0.25	0.60	0.37	−0.07	0.31	0.40	0.25
67		0.28	0.23	0.11	0.60	0.20	−0.14	0.24	0.25	0.23
99		**0.56**	0.49	0.36	0.69	0.46	−0.04	0.43	0.49	0.40
**Stage_4**	0.45**									
116		**0.49**	0.45	0.47	0.39	0.63	0.01	0.27	0.40	0.29
108		**0.58**	0.55	0.54	0.30	0.69	0.18	0.25	0.41	0.47
92		**0.50**	0.44	0.43	0.33	0.67	−0.04	0.28	0.40	0.44
28		**0.59**	0.62	0.58	0.32	0.67	0.08	0.30	0.39	0.49
52		**0.45**	0.43	0.32	0.34	0.72	0.00	0.19	0.36	0.29
**Stage_5**	0.45**									
69		0.04	0.01	0.09	−0.21	0.01	0.65	−0.20	−0.14	0.20
5		**0.46**	0.18	0.20	−0.01	0.16	0.71	0.01	0.01	0.32
109		**0.48**	0.11	0.13	0.06	0.02	0.61	−0.03	0.02	0.25
93		**0.47**	0.04	0.15	0.03	0.06	0.57	0.03	0.09	0.18
101		0.04	−0.03	0.13	−0.22	0.03	0.60	−0.17	−0.07	0.10
**Stage_6**	0.24**									
46		0.22	0.13	0.03	0.34	0.10	−0.20	0.64	0.31	0.05
86		0.24	0.17	0.03	0.22	0.04	−0.07	0.64	0.25	0.18
102		**0.47**	0.42	0.38	0.38	0.32	−0.11	0.60	0.44	0.35
38		**0.51**	0.38	0.25	0.39	0.35	0.05	0.63	0.41	0.48
6		0.31	0.21	0.13	0.39	0.14	−0.13	0.67	0.28	0.20
**Stage_7**	0.31**									
119		**0.52**	0.47	0.45	0.42	0.44	−0.03	0.40	0.63	0.31
47		**0.47**	0.42	0.39	0.38	0.34	0.05	0.35	0.57	0.33
39		**0.49**	0.42	0.33	0.36	0.42	−0.04	0.35	0.64	0.45
63		**0.41**	0.30	0.37	0.29	0.28	−0.01	0.33	0.66	0.32
7		**0.61**	0.57	0.42	0.55	0.49	−0.05	0.47	0.75	0.39
**Stage_8**	0.53**									
56		**0.58**	0.58	0.45	0.31	0.42	0.26	0.24	0.42	0.70
40		**0.50**	0.40	0.34	0.33	0.34	0.24	0.25	0.37	0.66
48		**0.65**	0.52	0.54	0.43	0.49	0.20	0.41	0.46	0.73
64		**0.41**	0.27	0.38	0.10	0.34	0.45	0.08	0.21	0.63
96		**0.48**	0.48	0.29	0.30	0.38	0.17	0.32	0.26	0.58

**p<0.01.

*Stage_1: The first stage (trust vs. mistrust); Stage_2: The second stage (autonomy vs. shame and doubt).*

*Stage_3: The third stage (initiative vs. guilt); Stage_4: The fourth stage (industry vs. inferiority).*

*Stage_5: The fifth stage (identity vs. role confusion); Stage_6: The sixth stage (intimacy vs. isolation).*

*Stage_7: The seventh stage (generativity vs. stagnation); Stage_8: The eighth stage (integrity vs. despair).*

*Eight stage: A composite score of the eight subscales. MCS, Mental component scale of SF-36.*

*The bold represented the values which were larger than 0.4.*

#### Validity of the Chinese Inventory of Psychosocial Balance Short-Form

Table 4 presents the correlations among each item of the subscale and other stages in the CIPB-SF. All subscales of the CIPB-SF had significant correlations with their own stage, and most of the correlation coefficients were > 0.4 except, item 67 (from the initiative stage), items 69 and 101 (from the identity stage), and items 46, 86, and 6 (from the intimacy stage). The three stages of initiative, intimacy, and identity had fair validity.

The eight subscales of the CIPB-SF and their total scores exhibited significant moderate to high positive correlations with their own stage. All of the items had significant positive correlations with their own stage, with greater correlation coefficient than others (Table 4).

#### Confirmatory Factor Analysis of the Chinese Inventory of Psychosocial Balance Short-Form

**FIGURE 2:**
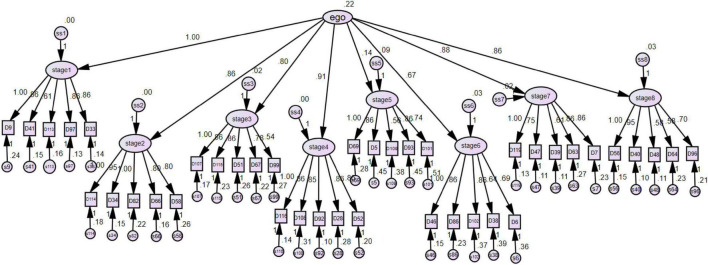
The model 1.

**FIGURE 3:**
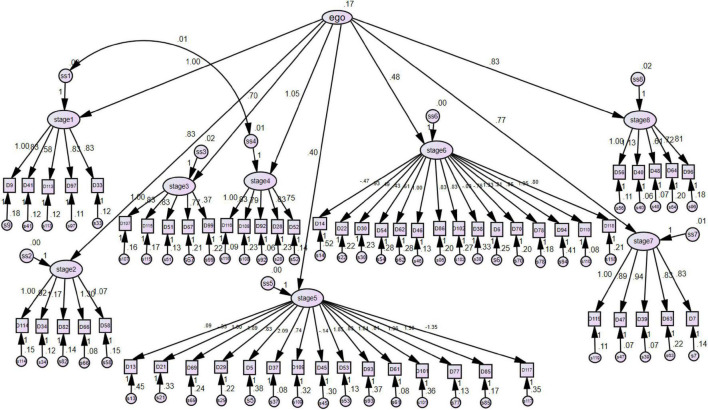
The model 2.

The aim of the CFA was to verify the models of questionnaires by structural equation (Olmedo Moreno et al., 2014). Based on the results of item analysis and factor analysis of the C-IPB, forty items were retained as the CIPB-SF and we defined those as Model 1 (Figure 2). The CIPB-SF we developed showed good reliability in all eight stages and appropriate validity, except the fifth and sixth stages. Therefore, the short-form fifth and sixth stages scale were replaced by the original fifth and sixth stages scale of IPB, and we defined those as Model 2 (Figure 3). Both the models will be evaluated by model fit index for assessment. As shown in Table 5, the measurement model showed an acceptable data fit included χ^2^/degrees of freedom, root mean square error of approximation (RMSEA), and Parsimony goodness-of-fit index (PGFI). However, the root mean square residual (RMR) was above the critical limit, 0.05, and the GFI was slightly less than the standard level. Sharma et al. (2005) indicated that the GFI was affected by sample size. The larger sample size should be considered in the future researches.

**TABLE 5:** Comparative summary of model fit.

	χ^2^	df	χ^2^/df	RMR	GFI	PGFI	CFI	TLI	RMSEA	AIC
Model 1	1501.21	746	2.01	0.09	0.76	0.69	0.82	0.53	0.05	1649.21
Model 2	3099.611	1715	1.81	0.10	0.67	0.62	0.62	0.61	0.05	3329.61

Akaike information criterion is used for model selection, and the model with lower value means that is better fit for the data. In the study, Model 1 is a better fit for the data compared to the Model 2 (Table 5).

## Discussion

In this study, we translated the IPB into Chinese to construct the CIPB-SF through factor analysis. The results obtained confirmed that the SC-IPB is a valid and reliable comprehensive tool that can be used to evaluate ego development among the Chinese elderly population. The previous studies indicated that the length of a questionnaire is important because it can directly affect response rates, survey costs, and data quality (Lavrakas, 2008 “Encyclopedia of Survey Research Methods,”). We shortened the 120-item IPB to a 40-item CIPB-SF with similar reliability and validity. In its ability to avoid any unnecessary interference from the questionnaire completion being a time-consuming task, the 40-item CIPB-SF can be considered superior to the original scale.

In this study, the CIPB-SF was found to have good reliability. Moreover, the Cronbach’s α of each of the eight subscales was between 0.81 and 0.89. We compared the reliability of the CIPB-SF with that of the IPB. The Cronbach’s α in the CIPB-SF was greater than that in the IPB. The values of the Cronbach’s α coefficients in the IPB were between 0.64 and 0.79 (Domino and Affonso, 1990). In addition, the highest Cronbach’s α coefficient value was observed in the industry stage and the lowest was observed in the intimacy stage of the IPB. In this study, the highest value of Cronbach’s α coefficients of the CIPB-SF was observed in the identity stage. Although the trust and integrity stages had the lowest Cronbach’s α coefficient values of the CIPB-SF, the Cronbach’s α coefficients of the eight stages of the CIPB-SF had similar values, ranging between 0.81 and 0.89. The autonomy stage (Cronbach’s α = 0.65) had the lowest Cronbach’s α value in the IPB.

The 30–35-day test–retest reliability in the subscale scores of the IPB were < 0.50 (Domino and Affonso, 1990). During the CIPB-SF development process, the subjects who participated in the study were anonymously recruited from the community. Therefore, assessing the test–retest reliability was not possible.

The validity of the IPB was assessed by examining the associations of the IPB subscales to the CPI–social maturity index (Hannah et al., 1996). Six of the eight IPB scales were found to have a positive significant correlation with the CPI-social maturity index, with only the autonomy and the intimacy stage exhibiting coefficients (Hannah et al., 1996). The CIPB–SF of the study had acceptable validity, except for the intimacy and identity stages. The validity testing results of the CIPB-SF were similar to those of the IPB. The reliability and validity of the identity stage differed between the two questionnaires, IPB and CIPB-SF. Cultural differences between the Eastern and Western versions were expected. The older adults who were a product of the Taiwanese experiment in Progressionism must have studied hard during their adolescence period to succeed in examinations for entering higher education. During their youth, these older adults had also witnessed the colonial rule of Japan. Therefore, they must have faced racial discrimination and unequal treatment in society. The “monomania to study medicine” and strong parental authority also emerged in their era (Chen, 2010). Cultural differences may have resulted in negative consequences, especially in the identity stage.

The intimacy vs. isolation stage had the lowest quality in the CIPB-SF in terms of convergent and discriminant validity. The CIPB-SF was developed on the basis of the IPB; however, the social backgrounds of these scales pertain to differ in terms of the expression of love. According to a study that explored Taiwanese male older adults through narrative inquiry, children born in Taiwan in the 1940s tend to have an initial life attitude of fatalism, but are not willing to yield to fate. This finding also highlights that men attach considerable importance to personal achievements and career development (Chuang and Lin, 2017). In view of the aforementioned culture differences, we recommend future researchers to perform another advanced study on the intimacy vs. isolation stage using qualitative research methods.

In CFA, the GFI values were 0.76 in Model 1 and 0.67 in Model 2. This result indicated that the model fit is only fair but not in good-fit. This may due to the translation process, under crossculture context, and reduction of the number in items. For instance, in Eastern, people are more conserved in showing intimacy and love than in Western culture. These factors may reduce the grade of model fit. This CIPB-SF could be tested and modified in future studies for developing a more suitable measurement tool based on Erikson theory.

To construct the IPB, 528 subjects, including students, adults, elderly individuals, and retired adults, between the ages of 21 and 88 years were included in the study (Domino and Affonso, 1990). A total of 308 subjects aged ≥ 65 years who participated in the development of the CIPB-SF were different from those recruited for the IPB. The IPB is a well-tested questionnaire, and the CIPB-SF was developed following the original questionnaire, which was first translated and then shortened. On the basis of Erikson’s theory and the IPB, scales pertaining to individual stages may also be used in isolation. Scales concerning individual stages that are tested in corresponding groups should be considered in the future.

Addressing the weaknesses of the present study, the first is the present research that used a cross-sectional design. Undoubtedly, it would be desirable to acknowledge the present study *via* applying a longitudinal study design. However, the IPB is already a well-tested questionnaire, and the present study focused emphatically on contributing to the Chinese-form questionnaire.

Then, the present study excluded the older adults who lived in long-term care institution. There was some discrepancy among older adults who lived in long-term care institution and community. Üzar-Özçetin and Ercan-Şahin (2020) indicated that older adults who have to live in nursing homes may feel deprived and isolated from society. To avoid the interference of the sampling, we recruited healthy Taiwanese elderly adults who were also independent. However, further research is needed to determine institutional residents’ validity or crosscultural validity.

The CIPB-SF can be used as a tool to investigate the status of ego development in the elderly community. Finally, future studies should implement and test CIPB-SF among different subjects that enhance the applicability and practicality of CIPB-SF.

## Conclusion

Erikson’s ego development theory is a rare psychosocial theory that encompasses the entire life span ranging from infancy to late adulthood. However, the lack of a credible Chinese version of the scale may impede research on ego development in Taiwan. This study constructed a 40-item CIPB-SF with good reliability (Cronbach’s α = 0.81–0.89) and validity. Due to its conciseness, the 40-item CIPB-SF was more appropriate to apply in for the Chinese elderly population to avoid physical overload. This scale can also serve as a useful tool for convenient screening in the future.

## Limitation

There was some limitation in this research. First, the sample size of this study was less than 500 subjects, yet the internal consistency test of CIPB-SF and the eight stages were still good (Cronbach’s α = 0.81–0.89). We will invite other research teams to participate in the study related to CIPB-SF in the future. Second, the low levels of CFA comparative indices (CFI, GFI, and TLI), although an absolute fit index like RMSEA were good. Although the comparative indices (CFI, GFI, TLI) indicated that the model fit was fair, Sharma et al. (2005) pointed out that the GFI was affected by sample size, and our sample size was less than 500, which is not perfect in SEM analysis. Further study will be needed to recruit multiple research teams to cooperate in crossdomain and crosscultural validity.

## Data Availability Statement

The raw data supporting the conclusions of this article will be made available by the authors, without undue reservation.

## Ethics Statement

The studies involving human participants were reviewed and approved by the Institutional Review Board (IRB) of China Medical University Hospital in Taiwan approved the study. The committee’s reference number is “DMR95-IRB-144.” Written informed consent was obtained from participants. The patients/participants provided their written informed consent to participate in this study.

## Author Contributions

P-YC wrote the main manuscript text, performed the research, and analyzed the data. CL interpreted the data. W-CH checked the statistical process. T-PY revised the sentence and checked the grammar. All authors reviewed the manuscript.

## Conflict of Interest

The authors declare the study received funding from China Medical University Hospital (Grant No. DMR-106-147). The funding body played a role in the design of the study and in writing the manuscript.

## Publisher’s Note

All claims expressed in this article are solely those of the authors and do not necessarily represent those of their affiliated organizations, or those of the publisher, the editors and the reviewers. Any product that may be evaluated in this article, or claim that may be made by its manufacturer, is not guaranteed or endorsed by the publisher.
